# Novel differential linear B‐cell epitopes to identify Zika and dengue virus infections in patients

**DOI:** 10.1002/cti2.1118

**Published:** 2020-02-19

**Authors:** Siti Naqiah Amrun, Wearn‐Xin Yee, Farhana Abu Bakar, Bernett Lee, Yiu‐Wing Kam, Fok‐Moon Lum, Jeslin JL Tan, Vanessa WX Lim, Wanitda Watthanaworawit, Clare Ling, Francois Nosten, Laurent Renia, Yee‐Sin Leo, Lisa FP Ng

*Clinical & Translational Immunology* 2019; **9**, e1118

**Correction to:** Clin Trans Immunol 2019; 8: e1066; doi: 10.1002/cti2.1066; published online 26 July 2019

The authors omitted to acknowledge one of the funding agency who supported their work. The corrected paragraph in the Acknowledgments should read as follows:

This work was supported by core research grants provided to the Singapore Immunology Network (SIgN) by the Biomedical Research Council (BMRC), A*ccelerate GAP funding [project number: ETPL/18‐GAP054‐R20H], and also partially supported by the BMRC A*STAR‐led Zika Virus Consortium Fund [project number: 15/1/82/27/001], Agency for Science, Technology and Research (A*STAR), Singapore. SIgN Immunomonitoring and Flow Cytometry platforms are supported by BMRC IAF 311006 grant and BMRC transition funds #H16/99/b0/011.

The authors apologise for this error.

